# The dosimetric impact of titanium implants in spinal SBRT using four commercial treatment planning algorithms

**DOI:** 10.1002/acm2.14070

**Published:** 2023-08-04

**Authors:** Chieh‐Wen Liu, Young‐Bin Cho, Anthony Magnelli, Lilyana Angelov, Ehsan H. Balagamwala, Samuel T. Chao, Ping Xia

**Affiliations:** ^1^ Department of Radiation Oncology, Taussig Cancer Institute Cleveland Clinic Cleveland Ohio USA

**Keywords:** algorithms, spinal implants, spine SBRT, treatment planning

## Abstract

To evaluate the dosimetric impact of titanium implants in spine SBRT using four dose calculation algorithms. Twenty patients with titanium implants in the spine treated with SBRT without density override (DO) were selected. The clinical plan for each patient was created in Pinnacle and subsequently imported into Eclipse (AAA and AcurosXB) and Raystation (CC) for dose evaluation with and without DO to the titanium implant. We renormalized all plans such that 90% of the tumor volume received the prescription dose and subsequently evaluated the following dose metrics: (1) the maximum dose to 0.03 cc (Dmax), dose to 99% (D99%) and 90% (D90%) of the tumor volume; (2) Dmax and volumetric metrics of the spinal cord. For the same algorithm, plans with and without DO had similar dose distributions. Differences in Dmax, D99% and D90% of the tumor were on average <2% with slightly larger variations up to 5.58% in Dmax using AcurosXB. Dmax of the spinal cord for plans calculated with DO increased but the differences were clinically insignificant for all algorithms (mean: 0.36% ± 0.7%). Comparing to the clinical plans, the relative differences for all algorithms had an average of 1.73% (−10.36%–13.21%) for the tumor metrics and −0.93% (−9.87%–10.95%) for Dmax of the spinal cord. A few cases with small tumor and spinal cord volumes, dose differences of >10% in both D99% and Dmax of the tumor, and Dmax of the spinal cord were observed. For all algorithms, the presence of titanium implants in the spine for most patients had minimal impact on dose distributions with and without DO. For the same plan calculated with different algorithms, larger differences in volumetric metrics of >10% could be observed, impacted by dose gradient at the plan normalization volume, tumor volumes, plan complexity, and partial voxel volume interpolation.

## 1 INTRODUCTION

In radiation therapy, the typical practice is to avoid radiation beams directly entering through the metal implants as recommended by TG 63, where the focus was on bulky implant such as the hip prosthesis. For patients with spine metastases, stereotactic body radiation therapy (SBRT) has been a prevalent treatment option, achieving fast pain relief, excellent local control, and low toxicity.^2^ In these patients, spinal prostheses are frequently present; choosing beams to avoid the prostheses is not feasible due to the concaved tumor shape that surrounds the spinal cord and the prostheses. For a bulky metal implant, when a broad radiation beam passes through it, a portion of the radiation beam is obstructed due to a strong attenuation from the implant. Downstream from the obstructed beam, a slightly increased scattering dose from the non‐obstructed portion of the beam is observed. For a small metal implant, the effects of strong attenuation and increased in‐scatter from the implant may negate each other, depending on the energy of the radiation as well as materials and volumes of the implants. For treatment planning systems(TPSs) or computed tomography (CT) scanners that can support images with 12‐bit depth, the Hounsfield unit (HU) ranges from −1024 to 3071, corresponding to a maximum physical density of 2.67 g/cm^3^. A typical metal implant (e.g., titanium) has a physical density of 4.45 g/cm^3^ with approximately 5000 HU. To accurately account for metal implants during treatment planning, manually assigning the correct physical density to the metal implants is necessary. Supported by measurements in phantoms with a single beam or parallel‐opposing beams, several studies have reported the dose perturbation by the spinal hardware.^3^, ^4^, ^5^, ^6^ Using Pinnacle dose calculation algorithm for a SBRT plan using step‐shoot intensity‐modulated radiation therapy (IMRT), Wang et al.^7^ found a ∼0.5 Gy dose decrease for seven cases with prescription dose of 24 Gy when manually overriding the physical density with the correct number. Using the Analytical Anisotropic Algorithm (AAA) algorithm from Eclipse planning system for one step‐shoot IMRT and five volumetric modulated arc therapy (VMAT) plans, Tang et al.^8^ found the differences in V95% to the PTV between density override (DO) and no‐DO calculations were within 1% for both titanium and cobalt‐chrome implants, while the differences to maximum dose in the spinal cord were 0.02–0.18 Gy for titanium implants and 0.06–0.25 Gy for cobalt‐chrome implants. In the present work, we selected 20 patients with spinal prostheses treated with VMAT SBRT to study dosimetric effect with and without DO. Furthermore, we re‐calculate these plans with four different dose calculation algorithms from three different TPSs. The aims of this paper are to investigate (1) whether manually assigning the appropriate density to the spinal prostheses can improve the accuracy of the dose calculation for spine SBRT using VMAT technique and (2) whether different dose calculations algorithms have a significant impact on the plan quality metrics.

## 2 METHODS

### 2.1 Patient plan characteristic

Thirty patients with titanium implants treated with spinal SBRT between the dates of 9/20/2019 and 2/19/2021 at our institution were identified. Twenty patients were selected for this study where the titanium implants were within the tumor volumes. As listed in Table 1, the average implant volume for this cohort of patients is 35 cc (ranged from 8.22 to 62. 34 cc), and the average implant volume inside the tumor volume is 7.4 cc (ranged from 0.15 to 30.9 cc). The treated vertebral levels are also listed in Table 1. The treatment planning CTs were scanned using a 1.5 mm slice thickness on a Philips Brilliance Big Bore CT scanner following a clinical spine SBRT protocol with a tube peak voltage of 120 kVp. The Philips metal artifact reduction algorithm for orthopedic implants, O‐MAR, was used to mitigate the metal artifact. The tumor volume and the spinal cord were drawn on T1‐ and short tau inversion recovery (STIR)‐weighted MRI scans that were rigidly fused with the planning CT. The spinal cord/cauda equina volume was contoured 5–6 mm above and below tumor volume.^9^ No planning organ‐at‐risk volume was taken into account. Table 1 shows the prescription, beam energy, tumor volume, metal implant volume, and metal implant volume within the tumor.

**TABLE 1:** Patient characteristics.

Patient	Treated level	Prescription (Gy)/Fractionation	Beam energy (MV)	Tumor volume (cc)	Implant volume (cc)	Implant ∩ tumor (cc)
**1**	L2	16/1	10 FFF	176.37	51.33	7.21
**2**	T9‐T10	18/1	6 FFF	101.37	42.26	0.15
**3**	T3‐T5	18/1	10 FFF	89.95	39.82	1.63
**4**	C3	18/1	6 FFF	36.62	8.22	1.55
**5**	T4‐T5	18/1	10 FFF	65.87	30.36	3.06
**6**	L3‐L4	16/1	10 FFF	227.58	27.02	2.99
**7**	T9‐T12	18/1	10 FFF	352.94	26.98	9.4
**8**	T12‐L1	18/1	10 FFF	205.84	33.58	2.46
**9**	L2‐L5	18/1	10 FFF	327.14	23.29	17.96
**10**	T4‐T7	18/1	10 FFF	343.79	46.98	23.87
**11**	L4	18/1	10 FFF	209.7	14.61	2.93
**12**	C6‐T2	18/1	6 FFF	114.95	17.79	6.9
**13**	C5‐T1	30/4	6 FFF	101.14	11.69	0.67
**14**	T3‐T5	30/4	10 FFF	55.61	44.72	1.01
**15**	T8‐T10	30/4	10 FFF	366.68	46.79	10.37
**16**	C5‐T1	30/4	6 FFF	85.77	16.8	8.92
**17**	L4‐S1	30/4	10 FFF	637.25	62.34	3.17
**18**	L3‐L5	30/3	10 FFF	367.21	47.96	5.37
**19**	T7‐T9	30/4	10 FFF	197.49	71.86	7.77
**20**	T7‐T10	27/3	10 FFF	438.83	43.36	30.9

### 2.2 Clinical plans

**FIGURE 1:**
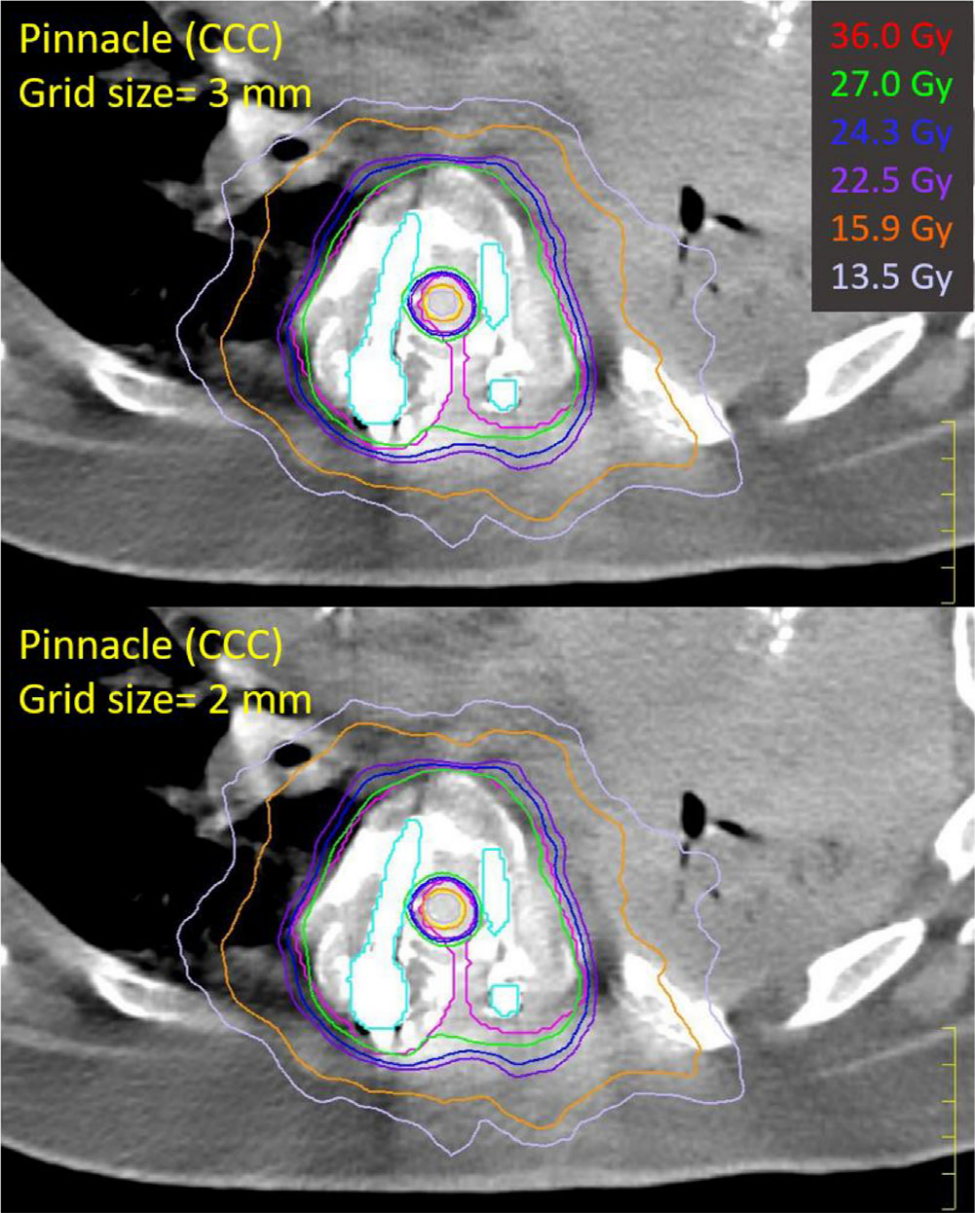
An example of dose distributions calculated with a grid size of 3 mm versus 2 mm for Patient 20. Magenta—tumor, yellow—spinal cord, cyan—spinal hardware.

All clinical plans were created in Pinnacle^3^ TPS (Philips Medical System, Cleveland, OH) for Varian Edge machines (Varian Medical Systems, Palo Alto, CA) with high‐definition MLC (2.5 mm leaf width) and 6 MV flattening filter free (FFF) or 10 MV FFF beams. These patients were planned without DO on the metal implants. Final dose was calculated using collapsed cone convolution (CCC) algorithm and a grid size of 3 mm or 2.5 mm. VMAT technique was used for all patients using 2−3 coplanar full arcs. Plans were optimized so that at least 90% of the tumor volume receives 100% of the prescription dose (*V*_100%RX_ > 90%). The institutional guidelines for treatment planning were followed based on the prescription dose. Per the recommendation of Task group 101,^10^ the use of grid sizes greater than 3 mm is discouraged for SBRT. However, using a grid size of 3 mm has been our clinical practice for spine SBRT plans for more than 10 years. We recalculated all twenty clinical plans using a dose grid of 2 mm. By using the same HUs as the clinical plans for the 2 mm dose grid recalculation plans, the mean relative difference of V_100%RX_ of the tumor was 1.03% ± 0.76% and the mean relative difference in the maximum dose to the spinal cord was −1.99% ± 2.30%. Figure 1 shows the dose distribution calculated with a grid size of 3 mm versus 2 mm of an example plan, in which the patient had the largest metal implant volume within the tumor. For a single fraction of 16 Gy or 18 Gy, planning constraints included <10% of the contoured spinal cord volume (L2‐3 and higher) receiving 10 Gy (*V*_10Gy_ < 10%) and the absolute maximum dose to the spinal cord of 14 Gy to a volume of no more than 0.03 cc (*D*_max_ < 14 Gy). For the cauda equina (below L2‐3), the planning goals were *V*_12Gy_ < 10% and *D*_max_ < 16 Gy. For three fractions, dose constraints were *D*_max_ < 22.5 Gy and *V*_15._9 Gy < 0.35 cc to the spinal cord and *D*_max_ < 25.5 Gy and V_21._9 Gy < 5 cc to the cauda equina. For four fractions, dose constraints were *D*_max_ < 25.6 Gy and *V*_18Gy_ < 0.35 cc to the spinal cord. Table 2 shows a summary of the volumetric spinal cord/cauda equina constraints in this study.^9^, ^11^

**TABLE 2:** Partial volume spinal cord/cauda equina constraints used in this study.

Fractionation	Planning goal
Spinal cord	
1	*V*_10Gy_ < 10%
3	*V*_15._9 Gy < 0.35 cc
4	*V*_18Gy_ < 0.35 cc
Cauda equina	
1	*V*_12Gy_ < 10%
3	*V*_21._9 Gy < 5 cc
4	*V*_26Gy_ < 5 cc

### 2.3 With and without density override (DO)

Because our clinical TPS (Pinnacle) only supports 12‐bit, the CT scale saturates at 4071 HU, corresponding to a mass density of 2.67 g/cm^3^ from our CT‐mass density (MD) calibration. Titanium has HU value of >8000, so during planning, automatic conversion of HU to MD is limited to materials with a mass density of 2.67 g/cm^3^. To accurately account for the MD to titanium in planning, a typical practice is to manually assign (override) the MD to the titanium contoured in the planning CT. For this reason, we created two plans for each patient with and without DO to the titanium alloy implant (4.42 g/cm^3^) in each of four dose algorithms evaluated in this study (described below). The total volume of the titanium hardware was delineated on the planning CT using a threshold automatic contouring tool in MIM (v7.2.7, Cleveland, OH). For each DO plan, the whole titanium hardware volume was manually assigned to 4.42 g/cm^3^. The plans with DO were set to have the same V_100%RX_ of the tumor.

### 2.4 Plan recalculation

We used four dose calculation algorithms to compare plans calculated with and without DO. We imported all clinical plans from Pinnacle into Eclipse (v15.1, Varian Medical System, Palo Alto, CA) and Raystation (v10B, RaySearch Laboratories, Stockholm, Sweden) using DICOM radiotherapy (RT) import for recalculation. The beam modeling data for the Edge machine used in Eclipse was obtained from one of our regional sites, while a generic Edge machine was used in Raystation, which was not clinically verified. To compared the basic beam characteristics, we collected the percentage depth dose (PDD) data for beam energies (6 MV FFF and 10 MV FFF for 10 × 10 cm^2^ field size) from Eclipse (AAA, AXB), Raystation (Collapsed Cone, CC), and Pinnacle (CCC). Compared with the data from Pinnacle, the differences among the systems were less than 0.5% (Table 3), indicating similar beam characteristics for 6 MV FFF and 10 MV FFF. The field size output factor comparison was shown in Table 4. Because the machine model for Eclipse (AAA and AXB) and for Raystation (CC) were not used clinically as well as different beam modeling methods in these TPSs, we were not able to verify other beam modeling parameters such as penumbra, head scattering, and MLC leave tips. To mitigate the beam modeling differences in these parameters, we chose to renormalize all recalculated plans to have 90% of tumor volumes receiving the corresponding prescription doses. For each patient, we imported CT images along with DICOM objects − RT‐structure, and RT‐plan exported from Pinnacle into Eclipse and Raystation. For each patient, a total of eight plans were generated with and without DO using Pinnacle (CCC), Eclipse (AAA and AXB), and Raystation (CC). The CT‐MD table used in Pinnacle (as seen in Table 5) was imported into both Eclipse and Raystation. Pinnacle (CCC), Eclipse (AXB) and Raystation (CC) requires a user‐defined CT‐MD calibration table, while Eclipse (AAA) requires a user‐defined CT‐relative electron density (RED) calibration table. The CT‐RED calibration table used in Eclipse (AAA) was obtained by the conversion factors determined from the International Commission on Radiological Protection publication 110 (ICRP‐110) phantom data.^12^ Dose calculation in Pinnacle (CCC) is reported as dose‐to‐medium. In Eclipse (AXB), while both dose‐to‐medium and dose‐to‐water are available, we choose the dose‐to‐medium mode for the calculation. In the AXB calculation, the metal hardware contour was assigned to material ‘titanium alloy’. In Eclipse (AAA) and Raystation (CC), a patient is modeled as water with varying density. Final dose calculation was performed using the same dose grid size (2.5 mm or 3 mm) matching with the clinical plans.

**TABLE 3:** Percentage depth dose data comparison.

	Pinnacle (CCC)	AAA		AXB		Raystation (CC)	
Depth (cm)^§^	Baseline	Calc.	Diff. (%)^†^	Calc.	Diff. (%)	Calc.	Diff. (%)
6 MV FFF							
1.4	100.00	99.84	−0.2	99.84	−0.2	99.53	−0.5
4.5	86.91	86.37	−0.5	86.37	−0.5	86.85	−0.1
7.0	75.63	75.16	−0.5	75.15	−0.5	75.74	0.1
10.0	63.51	63.19	−0.3	63.19	−0.3	63.49	0.0
16.0	44.20	43.84	−0.4	43.84	−0.4	44.00	−0.2
21.0	32.63	32.47	−0.2	32.47	−0.2	32.32	−0.3
10 MV FFF							
2.4	99.90	99.82	−0.1	99.82	−0.1	99.40	−0.5
5.5	88.45	88.40	−0.1	88.40	0.0	88.05	−0.4
8.0	78.47	78.40	−0.1	78.40	−0.1	78.22	−0.3
10.0	71.03	70.90	−0.1	70.90	−0.1	70.86	−0.2
15.0	55.30	55.40	0.1	55.40	0.1	55.16	−0.1
24.0	34.91	35.30	0.4	35.30	0.4	34.97	0.1

^§^ Field size 10×10 cm^2.^

^†^ Percentage difference is compared to the baseline (Pinnacle).

**TABLE 4:** Field size output factor comparison.

	Pinnacle	Eclipse		Raystation	
Field size (cm)	Baseline	Calc.	Diff. (%)^†^	Calc.	Diff. (%)
6 MV FFF					
3×3	0.846	0.841	−0.50	0.846	0.00
5×5	0.907	0.906	−0.10	0.907	0.00
10×10	1.000	1.000	0.00	1.000	0.00
15×15	1.051	1.049	−0.20	1.050	−0.10
10 MV FFF					
3×3	0.895	0.883	−1.20	0.891	−0.40
5×5	0.940	0.940	0.00	0.940	0.00
10×10	1.000	1.000	0.00	1.000	0.00
15×15	1.028	1.027	0.10	1.029	0.10

^†^ Difference is compared to the baseline (Pinnacle).

**TABLE 5:** CT to density table used in pinnacle.

CT number	Density
0	0.000
228	0.300
408	0.450
909	0.920
948	0.990
996	1.015
1012	1.045
1075	1.080
1091	1.150
1172	1.170
1443	1.340
1780	1.560
2219	1.840
8369	4.590
18233	10.000

### 2.5 Plan evaluation

To compare the dosimetric results with/without DO using four different calculation algorithms, we used the following dosimetric: maximum dose to 0.03 cc (*D*_max_), dose to 99% (*D*_99%_) and 90% (*D*_90%_) to the tumor; *D*_max_ and volumetric metrics of *V*_10Gy_, *V*_12Gy_, *V*_15.9 Gy_, *V*_21.9 Gy_, or *V*_18Gy_ (cc) of the contoured spinal cord/cauda equina.

## 3 RESULTS

### 3.1 With and without DO

**FIGURE 2:**
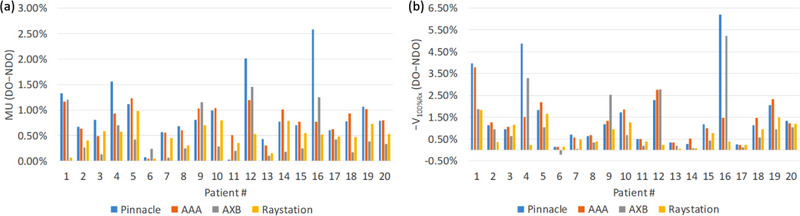
Dosimetric comparison compared between plans with (DO) and without (NDO) density override for different algorithms. (a) Relative MU difference by renormalizing plans to the same *V*_100%Rx_ of the target. (b) Difference (displayed in negative value) in V_100%Rx_ of the target using the same MU.

**FIGURE 3:**
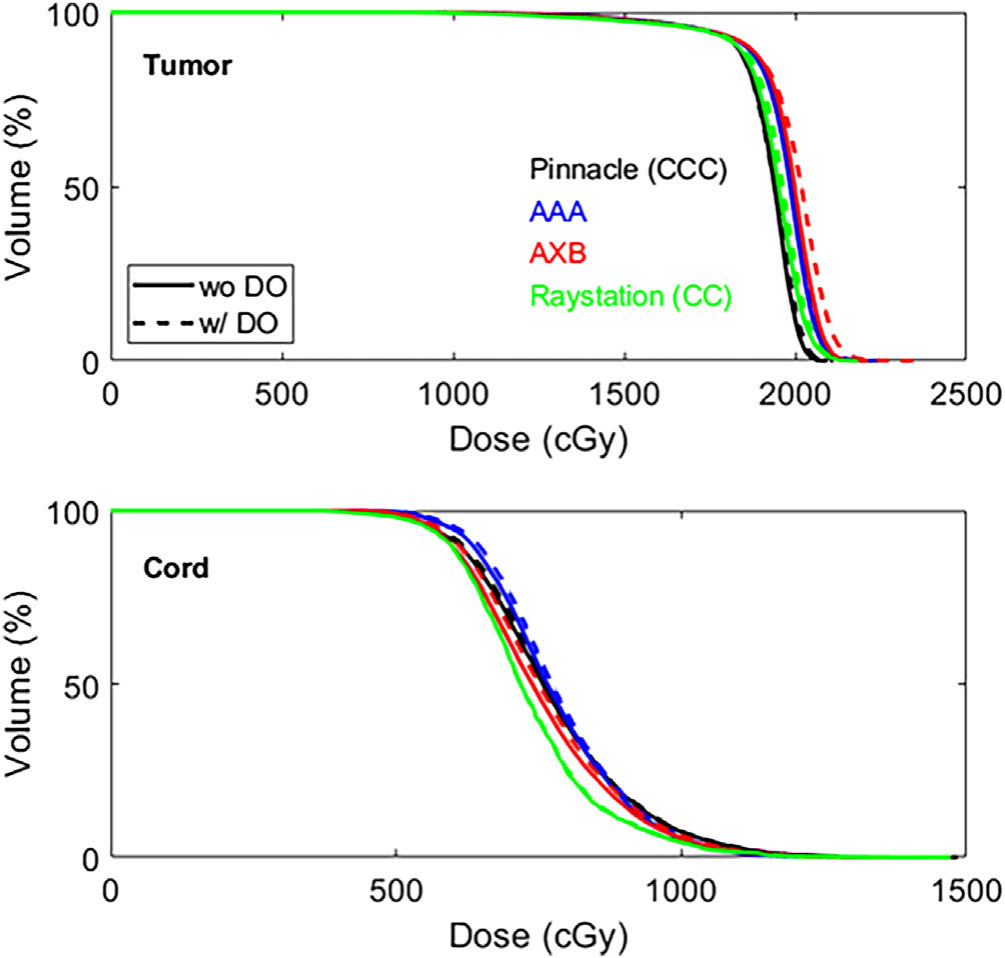
An example of DVH comparisons of the tumor and spinal cord for Patient 9.

To compare the impact of DO to the metal hardware within the same algorithm, we plotted Figure 2 using two different methods. In Figure 2a, we renormalized plans to achieve the clinical goal of V100%Rx of the target >90% after overriding the density of the implant. The relative MU differences after renormalization were <3%. In Figure 2b, we plotted the changes of V100%Rx of the target for plans recalculated with DO using the same MU for each plan. As shown in Figure 2b, V100%Rx could be reduced by up to ∼6% with DO to the implant. Overall, for most plans with/without DO, the differences in MUs were <1.5% or the differences in *V*_100%Rx_ were <3%. As described in the method section, we chose to renormalize all recalculated plans to achieve the same clinical goal of *V*_100%Rx_ of the tumor volumes >90% for the remaining analyses. Table 6 shows the results of the relative percentage difference of all metrics between with/without DO plans for each dose algorithm. The relative differences for all tumor dose metrics and spinal cord dose metrics were generally below 3.5% and 2.5%, respectively. For volumetric metrics of the spinal cord, that is *V*_XXGy_ (cc), we compared the absolute difference instead of relative difference because of the small contoured volume of the spinal cord. Differences in *V*_xxGy_ (cc) of the spinal cord between with/without DO plans were <0.12 cc. We observed the largest variation of 5.58% in *D*_max_ of the tumor in Patient 9 using AXB. Figure 3 shows the DVH comparisons of the tumor and spinal cord for this patient. Larger variations were observed in AXB compared with other algorithms. Overall, plans calculated with and without DO to titanium implants have minimal impact in dose distributions for all dose algorithms.

**TABLE 6:** Mean relative percentage difference of dosimetric metrics between plans with and without density override to titanium implants for each dose algorithm.

	Pinnacle (CCC)	AAA	AXB	Raystation (CC)
	Mean ± SD (range)	Mean ± SD (range)	Mean ± SD (range)	Mean ± SD (range)
*Tumor*				
∆Dmax (%)	0.20 ± 0.64 (−0.99–1.47)	0.87 ± 0.82 (−0.39–2.16)	1.60 ± 1.60 (−0.42–5.58)	0.09 ± 0.42 (−0.81–0.83)
∆D_99%_ (%)	0.11 ± 0.49 (−0.88–1.47)	0.06 ± 0.25 (−0.45–0.52)	0.48 ± 0.48 (−0.17–1.45)	0.03 ± 0.24 (−0.36–0.57)
∆D_90%_ (%)	−0.04 ± 0.30 (−0.28‐0.25)	0.00 ± 0.06 (−0.15‐0.15)	−0.02 ± 0.17 (−0.49 to 0.34)	−0.03 ± 0.17 (−0.06–0.30)
*Spinal cord*				
∆Dmax (%)	0.32 ± 0.83 (−0.99‐2.43)	0.17 ± 0.35 (−0.63–0.76)	0.76 ± 0.83 (−0.81–2.47)	0.18 ± 0.67 (−1.69–1.37)
∆ V_XXGy_ (cc)^§^	0.01 ± 0.02 (−0.03–0.05)	0.01 ± 0.05 (−0.10–0.10)	0.03 ± 0.05 (0.00–0.10)	−0.02 ± 0.03 (−0.12–0.00)

^§^ *V*_10Gy_, *V*_12Gy_, *V*_15.9 Gy_, *V*_21.9 Gy_, or *V*_18Gy_ (cc), depending on the prescription. Absolute difference is performed.

### 3.2 Dose calculation algorithm comparisons

**FIGURE 4:**
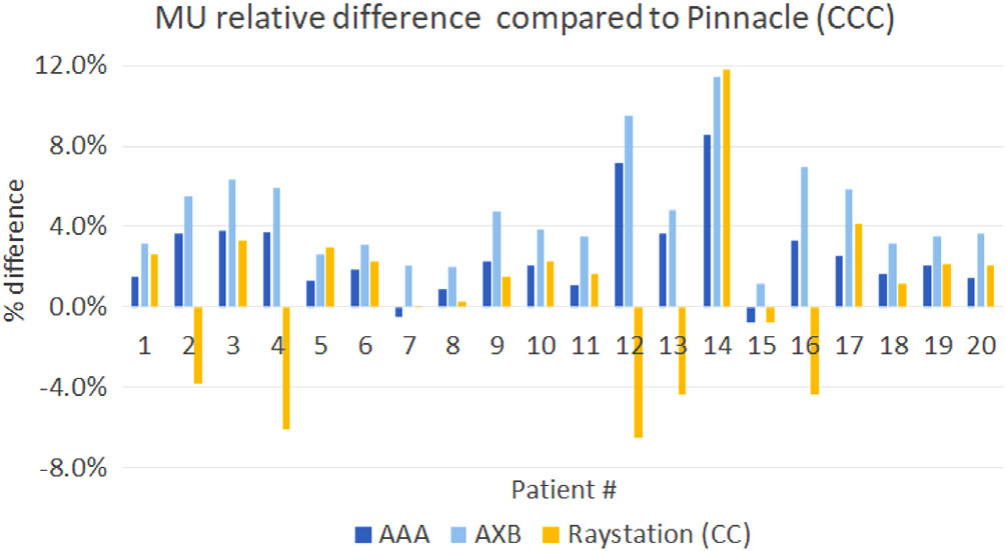
MU percentage difference of recalculated plans in AAA, AXB and Raystation (CC) compared with Pinnacle (CCC) plans.

Since DO to the titanium implant did not affect the dose distribution significantly, we compare the dose calculation among four algorithms without DO. Compared with corresponding Pinnacle plans, Figure 4 shows the relative difference in MU for the recalculated plans in AAA, AXB, and Raystation, defined as 100%*[MU(AAA, AXB or Raystation) −MU(Pinnacle)]/MU(Pinnacle). The relative difference in MU had a median of 2.41% for all recalculated plans. As shown in Figure 4, relatively large MU differences were observed in Patient 12 and Patient 14 for AAA, AXB, and Raystation. As shown in Table 1, patients 12 and 14 had treated with multiple vertebral bodies.

**FIGURE 5:**
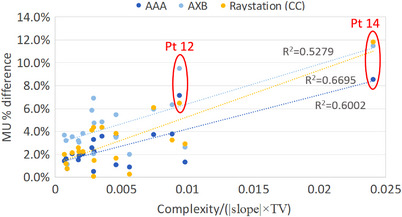
Relative difference in MU of recalculated plans in AAA, AXB and Raystation (CC) compared with Pinnacle (CCC) plans as a function of complexity/(|slope|×TV). Complexity is defined as monitor units (MUs) divided by the prescription dose (Rx). Slope is obtained at 90% of the volume receiving the prescription dose from the DVH of the tumor. TV is the tumor volume.

We speculate that such large difference in MUs could result from the plan complexity and dose gradient near the plan normalization point. Thus in Figure 5 we attempted to correlate the relative difference in MU with these quantities: complexity, slope, and tumor volume (TV). Complexity is defined as monitor units (MUs) divided by the prescription dose (Rx). Slope is obtained at *V*_100%Rx_ = 90% from the DVH of the tumor. A better correlation is shown when the MU difference is plotted against the combination of the three quantities [complexity/(|slope|×TV)], where we assume that the MU difference is proportional to the plan complexity and inversely proportional to the slope and the tumor volume. The plot explained why Patients 14 and 12 had larger percent difference in MU as they had the highest values of [complexity/(|slope|×TV)].

**FIGURE 6:**
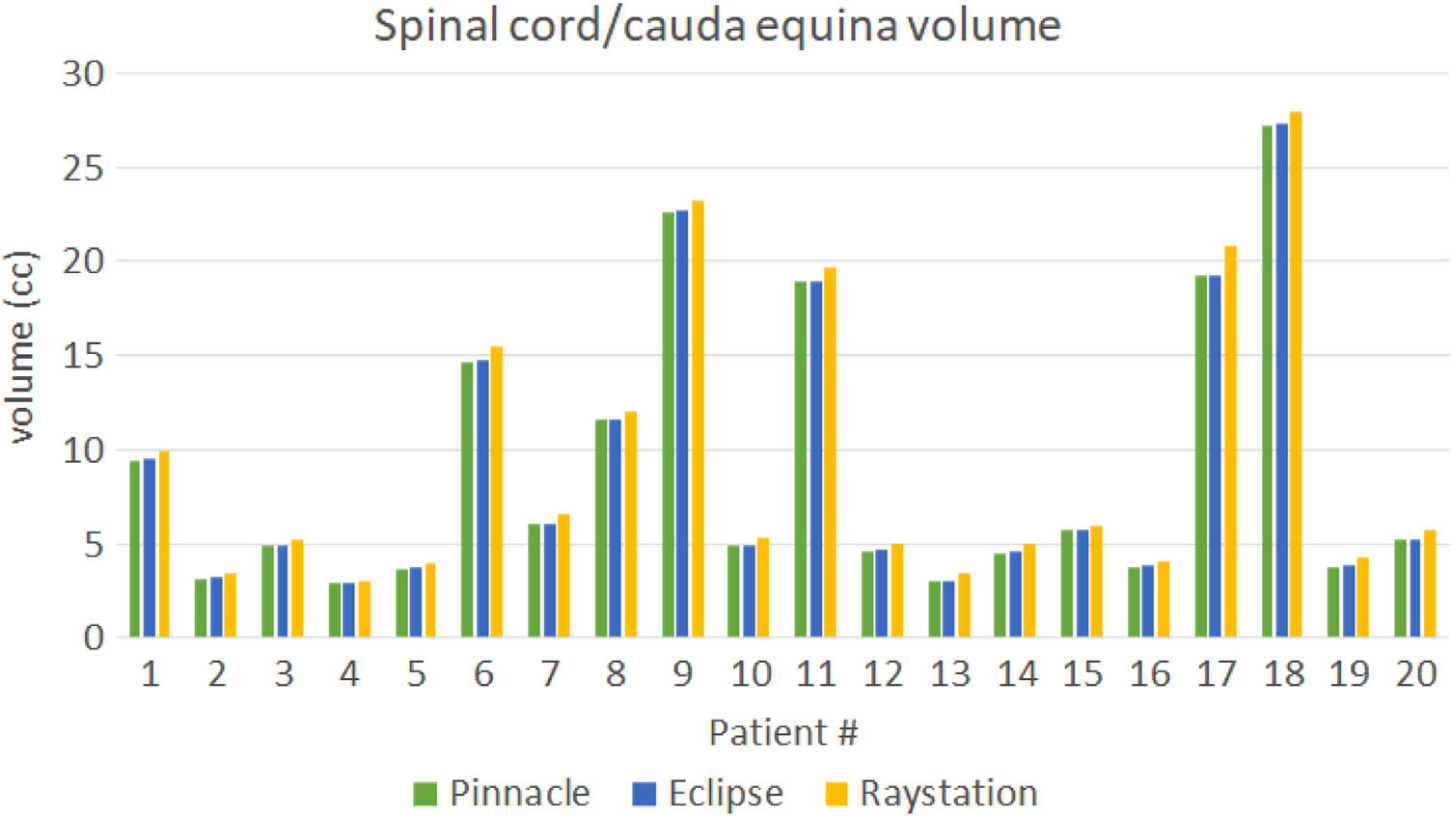
Spinal cord/cauda equina volumes reported in three different treatment planning systems.

**FIGURE 7:**
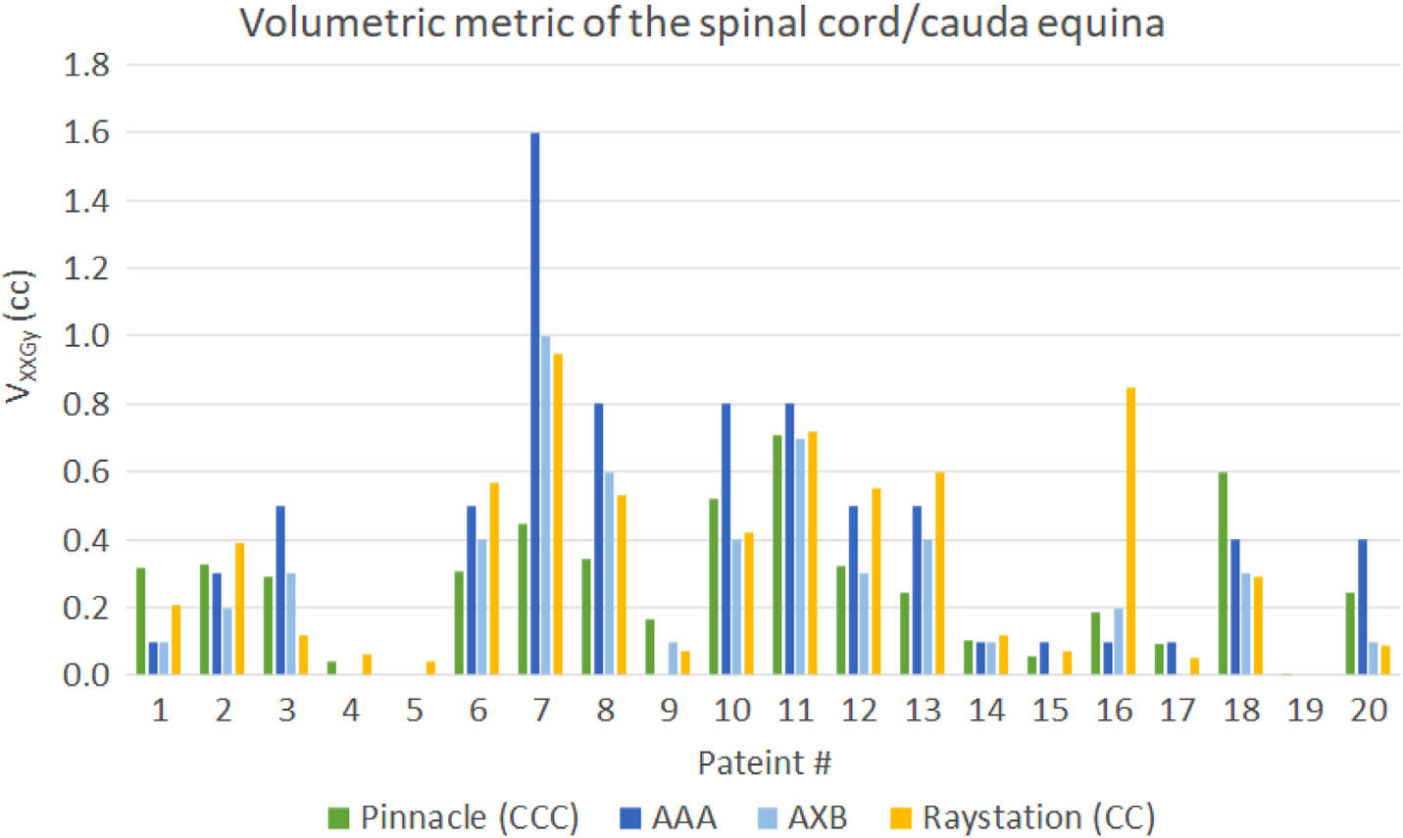
Partial volume constraints of the spinal cord/cauda equina of different dose calculation algorithms. *V*_10Gy_, *V*_12Gy_, *V*_15.9 Gy_, *V*_21.9 Gy_, or *V*_18Gy_ (cc) were reported based on the prescription.

**FIGURE 8:**
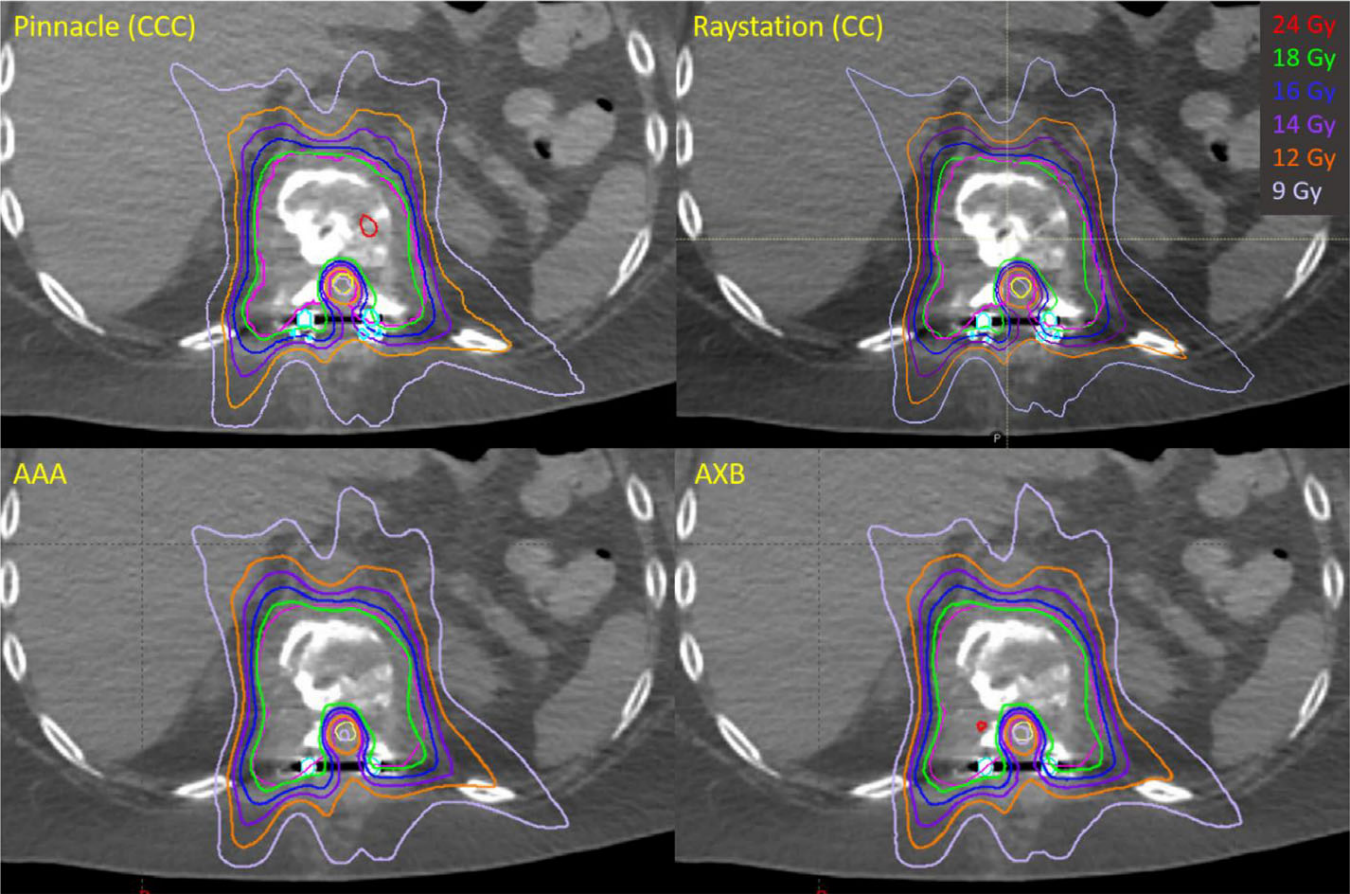
An example of dose distribution comparison among different algorithms for patient 7. The prescription dose is 18 Gy. Magenta—tumor, yellow—spinal cord, cyan—spinal hardware.

**FIGURE 9:**
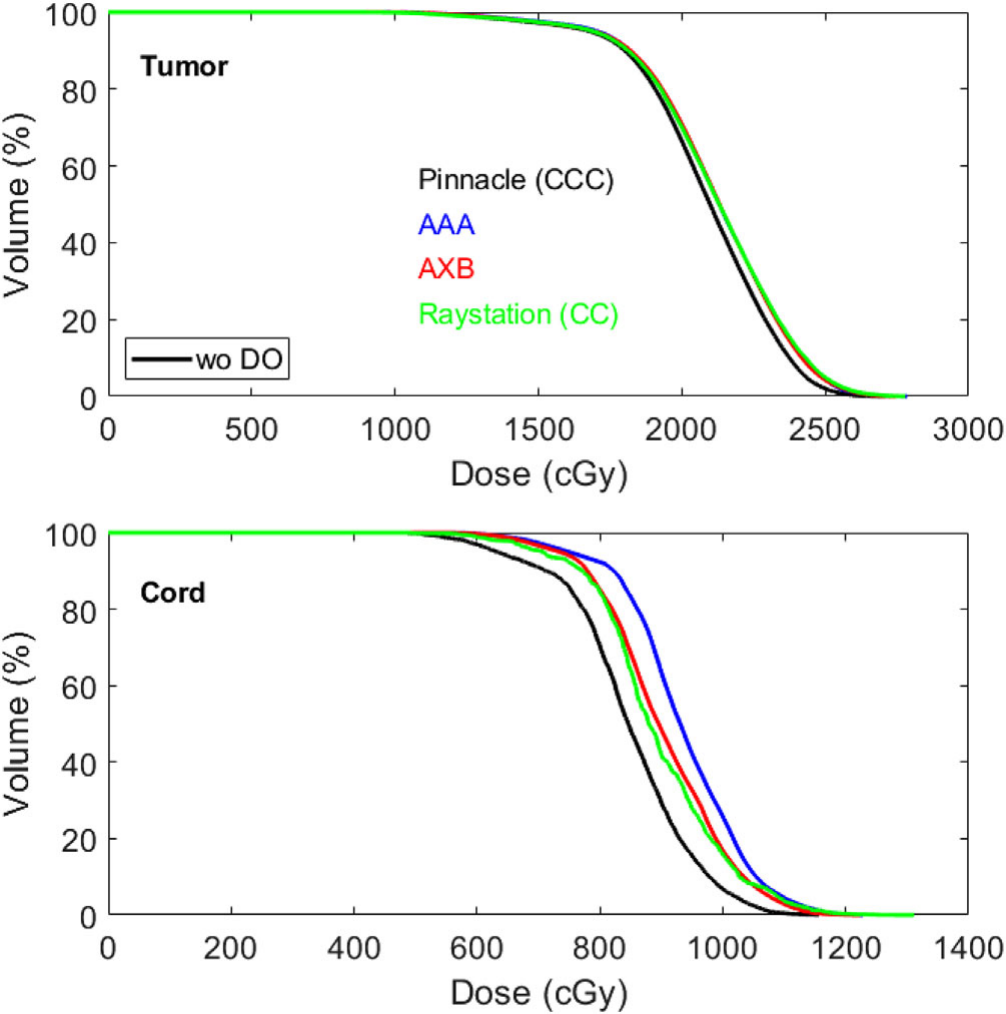
An example of DVH comparisons of the tumor and spinal cord for different dose calculation algorithms for patient 7.

Table 7 shows the dosimetric results of the tumor and the spinal cord from AAA, AXB, and Raystation (CC) plans in comparison with Pinnacle (CCC) plans. Since the plans were renormalized to have the same V_100%RX_ of the tumor, the differences in tumor dosimetric metrics between algorithms were relatively small. Therefore, we focused on the dosimetric comparisons of the contoured spinal cord/cauda equina. We plotted Figure 6 to show the differences in spinal cord/cauda equina volumes reported in different TPSs. Compared with Pinnacle, spinal cord/cauda equina volumes reported in Eclipse and Raystation showed smaller volumes as a consequence of the partial voxel volume interpolation, where the mean relative difference was −6.81% and −6.07%, respectively. Figure 7 shows *V*_XXGy_ of the spinal cord/cauda equina from different dose calculation algorithms for all patients. The largest deviation was seen in Patient 7 calculated with AAA, where the difference of *V*_10Gy_ compared with Pinnacle was 1.15 cc. The small spinal cord volume could be one of the reasons for such a difference. Another reason could be the high plan modulation, where MU/prescription was 12421/18 [MU/Gy]. Figure 8 shows the dose distributions among four different algorithms for this particular patient. The corresponding DVH comparisons of the tumor and the contoured spinal cord was shown in Figure 9. Overall, the values of *V*_XXGy_ shown in Figure 7 were inconsistent among four different dose calculation algorithms and the dose to the spinal cord was higher in AAA.

**TABLE 7:** Mean relative percentage difference of the dosimetric metrics for all patients compared with Pinnacle (CCC) plans.

	AAA	AXB	Raystation (CC)
	Mean ± SD (range)	Mean ± SD (range)	Mean ± SD (range)
*Tumor*			
∆Dmax (%)	3.63 ± 2.39 (0.31–7.82)	3.07 ± 1.99 (−0.06–8.00)	4.22 ± 2.51 (1.22–13.21)
∆D_99%_ (%)	2.31 ± 2.61 (−2.08–6.45)	1.23 ± 3.21 (−4.62–6.36)	−3.12 ± 3.79 (−10.36–3.01)
∆D_90%_ (%)	1.64 ± 0.83 (0.64–4.36)	1.70 ± 0.76 (0.63–3.67)	0.91 ± 1.05 (−0.03–4.54)
*Spinal cord*			
∆Dmax (%)	−2.07 ± 3.65 (−9.07–6.94)	−3.16 ± 3.74 (−9.87–4.08)	2.43 ± 5.37 (−7.23–10.95)
∆ V_XXGy_ (cc)^§^	0.11 ± 0.30 (−0.22–1.15)	−0.01 ± 0.18 (−0.30–0.55)	0.07 ± 0.24 (−0.31–0.67)

^§^ *V*_10Gy_, *V*_12Gy_, *V*_15.9 Gy_, *V*_21.9 Gy_, or *V*_18Gy_ (cc), depending on the prescription. Absolute difference is performed.

## 4 DISCUSSION

### 4.1 With/without DO

The first aim of this study is to evaluate the dosimetric impacts of DO to titanium implants in the spine for SBRT using VMAT technique in four dose calculation algorithms using the same treatment plan. For all algorithms, the presence of titanium implants in the spine had minimal impact on the dose distributions with and without DO.

Previous works have investigated the dosimetric impact of metal implants using phantom and patient datasets for spine metastasis and different disease sites.^13^ Wang et al.^7^ investigated the difference in dose calculation between plans with and without DO to the titanium implants using IMRT technique. They found that plans without DO overestimated the dose by a small amount (<1%) affecting 4% of the target volume for seven cases, which was believed to be minimal. In the study of six IMRT and VMAT paraspinal cases by Tang et al.,^8^ the PTV coverage was lowered by an average of ∼0.3% in DO for titanium compared with plans calculated without DO. Müller et al.^14^ reported that uncertainties in HU assignment of titanium alloy for spine patients showed no relevant difference in dosimetric quality for VMAT plans. These results are consistent with our findings, where dose calculations with and without manually assigning HU values to the titanium implants produced minimal difference in our VMAT plans. However, for some patients, for example, patient 16, the MU changes can be 2.6%. More TPSs are supporting 16 bits CT, which will include the corresponding HU number for titanium alloy. This study and other studies^7^, ^8^, ^13^ showed the common practice of not overriding titanium alloy during spine SBRT planning was clinically acceptable. However, if the implant was cobalt‐chrome, Tang et al.^8^ showed that manually density override was recommended even with 16 bits CT images and dose difference with and without DO was larger than those observed in titanium alloy. To prevent potential underdosage for some patients and for patients with cobalt‐chrome implants, we have changed our previous practice and adopted to use DO with appropriate densities in our institution.

### 4.2 Dose calculation algorithms

Dose algorithm calculation comparisons were of particular interest in heterogeneity and complex tissue interfaces, such as spine,^2^, ^5^, ^15^ lung,^16^, ^17^, ^18^ and HN.^19^, ^20^ Khan et al.^16^ reported that MUs required by AXB to deliver the prescribed dose were on an average 2% higher than AAA, which is similar to our results where MUs required to achieve the same tumor dose coverage in AXB were higher than AAA with a mean of 1.97%. Similar results were also seen in the study by Ong et al.,^17^ where a lower tumor coverage up to 8% was observed when calculated with AXB as compared with AAA using the same MUs for lung SBRT. In another lung SBRT study reported by Sarkar et al.,^18^ they found significant differences in dose distributions calculated with AXB and Raystation's Monte Carlo compared with other algorithms. We cannot conclude that AXB produces notable differences compared with the other three algorithms evaluated in our study. All parameters investigated by Sarkar et al. were within 5% between all algorithms, whereas the dosimetric endpoints in this study had differences of >5%. One reason could be the different disease sites being investigated. For example, the most pronounced difference between dose calculations in water versus in medium was seen in bone.^21^ Lower prescription doses in our study (16, 18 and 30 Gy) compared to those (48, 50, and 54 Gy) in their study could be another reason for the larger relative differences. Moreover, comparing to the study by Sarkar et al.^18^ where the analyses were compared in MIM, the dosimetric endpoints in this current work were obtained in each TPS, which avoided further partial volume interpolation in a different software. Maximum dose in the spinal cord in our study was on average lower by 1.17% in AXB than AAA, which is consistent with the results of the spine study by Hughes et al.,^15^ where they found the plans calculated in AXB were on average 4.5% colder than AAA when comparing the maximum dose in the spinal cord. Overall, comparing to our clinical plans, the relative differences for all algorithms had an average of 1.73% (−10.36% to 13.21%) for the tumor metrics and −0.93% (−9.87%−10.95%) for Dmax of the spinal cord. These results indicated that patients planned with different dose calculation algorithms may receive different target dose coverages/doses to normal tissues, impacted by dose gradient at the plan normalization volume, tumor volumes, plan complexity, and partial voxel volume interpolation.

### 4.3 Partial volume effect

Apart from the difference between dose calculation algorithms, volume calculation is handled differently in different TPSs.^19^, ^22^, ^23^, ^24^, ^25^ The volumes from the same DICOM structure set are reported differently because each TPS has its own algorithm to model 3D volumes, such as shape‐based interpolation and linear interpolation. DVH is calculated by binning the dose values inside the volume of specific structure through a sampling technique. These interpolation and sampling methods as well as different calculation algorithms result in variations in DVH statistics. Moreover, small structures were reported to have large deviation in DVH calculation especially in regions with a high dose gradient.^26^ As a consequence, when evaluating endpoints that have smaller volumes such as *V*_XXGy_ (cc) to the contoured spinal cord, larger variations across different TPSs are likely to be expected. Note that we typically evaluate the contoured spinal cord (not the entired spinal cord) using the volumetric metric, that is *V*_XXGy_ (%), in our clinical practice. The reason we chose to analyze *V*_XXGy_ in absolute volume (cc) in this paper is that larger differences were observed in percentage volume due to small *V*_XXGy_ and the spinal cord volumes.

### 4.4 Dose grid size

One limitation in this study is that we did not test the effect of grid size while using our clinical practice of a 3 mm grid size. The second aim of this study is to compare other algorithms to our clinical system (Pinnacle (CCC)) using the same dose grid sizes as those in the clinical plans. In our clinical practice, we have used a 3 mm dose grid size for 10 years and achieved excellent clinical outcomes as well as high IMRT/VMAT QA passing rates. The question is whether our results are due to the use of a relatively coarse dose grid size. In the study by Wang et al.,^7^ they found that dose perturbations by the titanium rod decreased by half when the gird size increased from 2 to 4 mm. In the present work, the majority of the plans was calculated using a 3 mm gird size, so dose perturbations by the titanium implants might be less pronounced. By recalculating 20 clinical plans with 2 mm dose grid, we found a small changes in V_100%RX_ of the tumor about 1.03% ± 0.76%. Thus, we believe the use of 3 mm dose grid did not alter our results significantly. Our findings are supported by the study of the effect of the grid size in spine SBRT.^27^ Snyder et al.^27^ found an average difference of <1.2% for all dosimetric indices using 1 mm, 1.5 mm and 2.5 mm grid sizes, while a maximum difference of 5.9% in Dmax of spinal cord was observed when using a grid size of 1 mm.^27^

### 4.5 Same MUs or same tumor dose coverage

In our study, we compared methods of having each recalculated plan normalized to the same dose coverage to the tumor volume for each algorithm and having the same MU for each recalculated plan to assess dose coverage to the tumor targets. We believe both methods have advantages and dis‐advantages. Because inherent differences in the beam models among the three TPSs, we chose to normalize all plan to have the same tumor dose coverage while comparing the spinal cord dose metrics because most spine SBRT plans are near the limit of spinal cord dose constraints. Other studies used our approach of normalizing to the same tumor dose coverage. In the paper published by Sarkar et al.,^18^ plans re‐calculated with different algorithms were all normalized to the same dose coverage of the tumor volume. We believe either method is justified for such comparisons since there is no analytical model involved that serves a ground truth^23^ for the aforementioned and the current studies. Here lies the limitation of this study. The treatment machine modeled in the three TPSs are not the same machines despite they are from the same type of machine (i.e. Edge, Varian). The subtle differences in machine characteristics and differences in beam modeling from the different TPSs may contribute to the observed differences in MUs and or tumor volume coverages. A future study will be to use a phantom measurement to establish the ground truth.

## 5 CONCLUSION

For all algorithms investigated in this study, the presence of titanium implants in the spine had minimal impact on dose distributions between plans with and without DO to the metal implants. For TPS comparisons using the same plan, larger differences of >10% were observed. Dose variations in different TPSs presumably arise from inherent differences in dose calculation algorithms, but dose gradient at the plan normalization volume, structure volumes, plan complexity and partial voxel volume interpolation should also be considered when doing evaluation.

## AUTHOR CONTRIBUTIONS

Conception and design: Chieh‐Wen Liu, Ping Xia; Data collection: Chieh‐Wen Liu, Anthony Magnelli; Data analysis and interpretation: Chieh‐Wen Liu, Young‐Bin Cho; Manuscript writing: Chieh‐Wen Liu, Ping Xia; Final approval of manuscript: Lilyana Angelov, Ehsan H. Balagamwala, Samuel T. Chao, Ping Xia.

## CONFLICTS OF INTEREST STATEMENT

The authors declare no conflict of interest.
